# Design and Fabrication of Biomimetic Gradient Bone Tissue Engineering Scaffolds: Evolution from Single-Gradient to Multi-Gradient

**DOI:** 10.3390/gels12020131

**Published:** 2026-02-02

**Authors:** Haitao Liu, Junjun Liu, Chenhui Sun, Yuhan Wang, Yazhou Sun, Xiaoquan Shi

**Affiliations:** Department of Mechanical Engineering and Automation, Harbin Institute of Technology, Harbin 150001, China; hthit@hit.edu.cn (H.L.); 24s108275@stu.hit.edu.cn (J.L.); hdsunchenhui@163.com (C.S.); wangyuhandsot@163.com (Y.W.); sunyzh@hit.edu.cn (Y.S.)

**Keywords:** bone tissue engineering, gradient scaffolds, additive manufacturing, biofabrication, biomimetic design

## Abstract

The regeneration of bone and the repair of large segmental bone defects represent critical challenges in regenerative medicine. Natural bone tissue is an anisotropic material characterized by an intricate gradient distribution in structure, mechanical properties, and biochemical composition; this multi-dimensional heterogeneity is crucial for maintaining its physiological functions and guiding regeneration. Although tissue engineering scaffolds have demonstrated significant potential in the treatment of bone defects, homogeneous or single-gradient scaffolds often struggle to precisely recapitulate the high degree of heterogeneity and anisotropy of natural bone from the macroscopic to the microscopic level, thereby limiting their capability in repairing complex bone defects. In recent years, biomimetic gradient scaffolds—particularly those employing multi-gradient synergistic designs that integrate physical structure, biochemical composition, and mechanical properties—have emerged as a research frontier in this field due to their ability to accurately mimic the natural bone microenvironment and regulate cellular behavior. This research aims to systematically review the latest research progress in gradient scaffolds for bone tissue engineering. First, gradient characteristics of biomimetic gradient bone scaffolds are summarized; second, the design strategies for gradient scaffolds are discussed in depth, with a focus on the applications and advantages of advanced fabrication techniques, such as additive manufacturing, in constructing multi-dimensional gradient structures; finally, based on current research findings, the emerging development trends and future research directions of biomimetic gradient bone scaffolds are outlined to provide a reference for innovative breakthroughs in the field of bone tissue engineering.

## 1. Introduction

As an indispensable core supporting and functional regulatory organ of the human body, bone tissue serves not only as the structural cornerstone for maintaining morphology, bearing loads, protecting viscera, and facilitating movement, but also as a physiological hub for constructing the hematopoietic microenvironment, regulating calcium and phosphorus metabolism, and participating in immune modulation [1]. The continuity, integrity, and functional stability of the skeleton directly determine the body’s locomotor capacity and internal environmental balance, forming the core foundation for sustaining life activities and health quality. With the increasing incidence of various skeletal diseases and injuries, the clinical repair of bone defects has become a central challenge in the fields of orthopedics and regenerative medicine [2,3,4]. Globally, bone defect cases caused by trauma, bone tumor resection, deep infection, congenital deformities, and osteoporotic fractures are showing an upward trend year by year [5,6,7]. Statistics indicate that approximately 1.71 billion people worldwide suffer from musculoskeletal conditions—including low back pain, neck pain, fractures, other injuries, osteoarthritis, amputation, and rheumatoid arthritis [6]—with about 1.5 million severe bone infection cases annually involving the risk of amputation or even death in certain instances [8]. Although bone tissue possesses self-healing capabilities, it can only regenerate and remodel small defects/injuries (<6 mm); it remains insufficient for repairing larger defects [9]. This disruption of structural integrity is not only accompanied by persistent severe pain but may also lead to the loss of corresponding tissue or organ functions, severely affecting the patient’s quality of life and imposing a heavy burden on families and society [10]. The traditional method for treating bone defects is bone grafting [11], including autologous grafting, allogeneic grafting, and artificial bone grafting [12]. Autologous grafting is regarded as the “gold standard” for treating bone defects due to its superior osteoinductive capacity, low immunoreactivity, and excellent osteogenic performance [13]; however, its application is constrained by issues such as local infection and limited donor supply. Meanwhile, the application of allogeneic grafting is restricted due to immune rejection reactions and other factors [14].

Since the early 1990s, the emergence of bone tissue engineering scaffolds has provided a new avenue for solving the difficulties of bone defect repair [15,16]. The core logic lies in constructing functional three-dimensional structures through biomimetic design to simulate the biochemical properties of the extracellular matrix (ECM), thereby providing a 3D growth microenvironment for cells to guide tissue regeneration and compensate for the limitations of natural bone repair capabilities. The field of tissue engineering emphasizes that an ideal biological scaffold should not only provide physical space for cell adhesion, proliferation, and differentiation but also ensure the efficient transport of nutrients and metabolic wastes, while matching the mechanical properties of the native tissue [17,18]. Natural bone tissue is not a single homogeneous entity, but rather a complex system exhibiting high heterogeneity and anisotropy across multiple dimensions. It possesses a complex gradient structure, with distinct mechanical, compositional, and porosity gradients throughout its internal architecture—from cortical bone to cancellous bone, and from vascular elements to the bone matrix. This natural gradient structure endows bone tissue with excellent overall mechanical performance and local biological functionality. In the early stages of tissue engineering, scaffold designs were predominantly characterized by homogeneous compositions or uniform structures, resulting in intrinsic limitations in mimicking the critical heterogeneity and functional regionalization of bone tissue. Consequently, they failed to simultaneously meet the distinct biological and mechanical requirements on both sides of the interface, making it difficult to achieve the gradient characteristics in mechanical properties, pore structure, and compositional distribution found in actual bone tissue from cortical to cancellous regions. To address such issues, the tissue engineering field began to develop scaffolds with gradients, introducing continuous or discontinuous gradient changes at the macro, micro, or nano scales to precisely mimic the structure and function of natural bone. Research by numerous scholars has achieved transitions from high stiffness to low stiffness [19], optimized the transport of nutrients and metabolic wastes [20], and accomplished the guidance of critical cellular behaviors such as osteogenic differentiation or angiogenesis [21]. These studies have demonstrated that gradient scaffolds exhibit superior mechanical properties, biocompatibility, and bioactivity compared to homogeneous scaffolds, holding significant value for advancing the field of tissue engineering.

Despite the significant progress made by introducing single gradients in simulating local characteristics of bone tissue, natural bone tissue (especially the osteochondral interface, etc.) exhibits highly complex, multi-dimensional synergistic gradient characteristics [5,22]. Changes in mechanical strength are accompanied by synchronous variations in biochemical composition, micro-topological structure, and even the immune microenvironment. Relying solely on singular variations in physical structure or material composition is often insufficient to meet complex mechanical transmission requirements and to precisely regulate specific cell differentiation. Therefore, to further improve the efficiency and quality of bone defect repair, research hotspots in the tissue engineering field have recently converged on biomimetic bone scaffolds integrated with multi-gradients. Concurrently, with the rapid development of advanced manufacturing technologies such as additive manufacturing, electrospinning, and microfluidics [3], precise regulation of the scaffold’s internal structure has become possible, providing solid technical support for the design and fabrication of multi-gradient scaffolds. Researchers have begun dedicating efforts to simultaneously achieving multi-dimensional gradient changes in pore structure, mechanical strength, chemical composition, and even bioactive factor concentration within biological scaffolds [23,24]. This multi-dimensional gradient design at the physical, mechanical, and biochemical levels aims to more accurately mimic the natural bone tissue microenvironment, thereby meeting more complex mechanical performance requirements and achieving fine-tuned regulation of cellular behavior. Consequently, systematically categorizing the gradient characteristics of natural bone tissue, along with the design strategies, fabrication methods, and development trends of multi-gradient biomimetic bone scaffolds, is of great significance for promoting the functional design and clinical translation of bone tissue engineering scaffolds.

Against this background, this review focuses on the research progress of gradient bone tissue engineering scaffolds, systematically presenting their gradient characteristics, scaffold design methods, fabrication processes, and development prospects. First, the key gradient characteristics of biomimetic gradient bone scaffolds are systematically summarized. Second, emphasis is placed on elucidating the design methods, manufacturing processes, and approaches for constructing multi-gradients in bone scaffolds; the unique advantages and representative research findings of different design strategies, manufacturing technologies, and gradient construction methods are deeply scrutinized to clarify the core advantages of gradients in regulating bone regeneration. Finally, based on existing research findings, the emerging development trends and future research directions of biomimetic gradient bone scaffolds for tissue engineering are envisioned, providing a reference for innovative breakthroughs in the field of bone tissue engineering.

This review provides a comprehensive analysis of biomimetic gradient bone scaffolds, focusing on the integration of gradient characteristics, design strategies, and advanced fabrication processes. Section 2 systematically categorizes the fundamental gradient characteristics of biomimetic scaffolds, including geometric structural, material compositional, and crosslinking density gradients. Section 3 explores design methodologies, detailing specific strategies for geometric and material gradients while emphasizing the shift toward synergistic multi-gradient design methods to better mimic natural bone heterogeneity. Section 4 provides an in-depth discussion on fabrication processes, covering key techniques such as 3D bioprinting and electrospinning, as well as precise gradient construction methods like multi-nozzle extrusion and coaxial printing. Finally, Section 5 summarizes current research findings and outlines future perspectives. By systematically reviewing the entire workflow from gradient characteristic analysis to fabrication implementation, this review aims to provide insights for the development of next-generation functionalized bone tissue engineering scaffolds.

## 2. Gradient Characteristics of Biomimetic Bone Scaffolds

**Figure 1:**
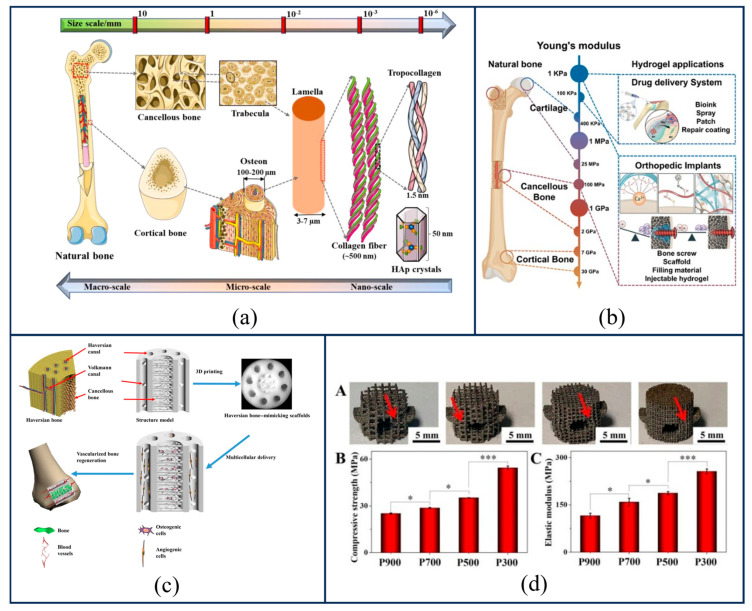
Characteristics of natural bone and bone scaffolds: (**a**) The hierarchical structure of natural bone, illustrating the organization from macroscopic cortical bone down to nanoscopic collagen fibers [25]; (**b**) Variation in the Young’s modulus of natural bone, highlighting the mechanical heterogeneity across different anatomical locations [26]; (**c**) Haversian scaffolds designed with a biomimetic multichannel architecture to replicate the native osteon system [27]; (**d**) Effect of pore size on the elastic modulus of scaffolds, demonstrating a decrease in mechanical strength as pore diameter increases [28].

Natural bone tissue is a hierarchical composite connective tissue comprising cells, fibers, and a matrix; as a critical component of the human body, it primarily performs functions such as mechanical support, organ protection, and hematopoiesis. It exhibits distinct and complex structural gradients [25,26] (as shown in Figure 1a) and mechanical gradients (as shown in Figure 1b) within its internal architecture and across its interfaces. These natural gradient structures endow bone tissue with superior global mechanical properties and localized biological functionality. To ensure that bone scaffolds possess properties matching those of the native tissue, it is essential to recapitulate the gradient structure of natural bone as closely as possible by introducing corresponding gradient variations into the scaffolds. Generally, gradients in bone scaffolds can be categorized into geometric structural gradients, material compositional gradients, and crosslinking density gradients; a detailed introduction to these three gradient characteristics follows below.

### 2.1. Geometric Structural Gradients

Geometric structural gradients in bone scaffolds aim to recapitulate the complex spatial heterogeneity of natural bone tissue at both the macroscopic morphological and microscopic topological levels. Researchers achieve gradient construction primarily by modulating geometric attributes such as porosity gradients, pore size gradients, and pore geometries, which play a critical role in facilitating cell survival and promoting tissue growth [29].

A porosity gradient refers to a continuous or stepwise variation in the volume percentage of voids along a specific direction of the scaffold. Higher porosity correlates with higher permeability; consequently, regions of high porosity significantly enhance scaffold permeability, providing ample space for deep cellular infiltration and the transport of nutrients and metabolic wastes [30]. Conversely, regions of low porosity serve as the primary load-bearing units, endowing the scaffold with the mechanical strength necessary to withstand physiological loads. Although high porosity typically results in a sharp decline in modulus and strength, gradient optimization strategy allow for the local optimization of bioactivity while simultaneously preserving the mechanical integrity of the overall structure. In current research, numerous scholars have successfully fabricated scaffolds with varying porosities using diverse methods. Advances in additive manufacturing have enabled the fabrication of complex gradient structures; for instance, Zhang et al. [27] successfully fabricated Haversian scaffolds integrating hierarchical Haversian bone structures via Digital Light Processing (DLP) 3D printing, as shown in Figure 1c. By altering the parameters of these Haversian biomimetic structures, the compressive strength and porosity of the scaffolds could be effectively modulated, achieving precise control over both porosity and mechanical performance.

A pore size gradient refers to the spatial variation in pore size distribution; this parameter directly dictates the specific surface area of the scaffold, thereby significantly influencing cell adhesion, proliferation, and differentiation behaviors [31]. Scaffolds with smaller pore sizes possess larger specific surface areas, which facilitate cell adhesion [32], whereas scaffolds with larger pore sizes favor cell migration [31] and exhibit superior permeability and biocompatibility. Although large-pore structures are often accompanied by a compromise in mechanical strength (as shown in Figure 1d) [28], the introduction of gradient allows for the preservation of local large-pore transport channels while maintaining the overall mechanical integrity of the structure. Regarding the specific biological effects of pore dimensions, a study by Chen et al. [33] provided compelling evidence demonstrating that smaller pore sizes and lower porosities promote the adhesion, proliferation, and osteogenic differentiation of rat bone marrow mesenchymal stem cells (rBMSCs). This suggests that in scaffold design, utilizing small-pore regions to induce osteogenic differentiation while employing large-pore regions to promote tissue ingrowth represents an effective strategy for achieving the comprehensive optimization of the bone repair process.

In addition to porosity and pore size, pore geometry constitutes a critical dimension for regulating scaffold properties. Common pore shapes typically include triangular, circular, and square geometries. Furthermore, in recent years, Triply Periodic Minimal Surface (TPMS) structures described by mathematical functions (such as Gyroid and Diamond configurations) have been demonstrated to outperform traditional truss or polyhedral structures in balancing permeability with mechanical modulus, owing to their zero mean curvature and highly interconnected pore networks [34]. Variations in these microscopic geometric features do not merely alter the physical appearance of the scaffold; more importantly, they influence mechanical stability and biological effects by modulating local curvature and stress distribution states. Cells possess the ability to regulate their cytoskeletal reorganization and migration patterns through curvature-sensing mechanisms [35]. On this basis, a study by Lu et al. [36] provided an in-depth elucidation of this mechanism, revealing that Diamond-type scaffolds, by influencing the adhesion state of Bone Marrow Mesenchymal Stem Cells (BMSCs), can activate higher levels of RhoA/ROCK2 signaling pathway transduction, thereby enhancing the migration capability and osteogenic differentiation potential of BMSCs. However, research by Hollister et al. [37] is noteworthy; they observed that after a period of implantation, there was no statistically significant difference in bone ingrowth between scaffolds with circular pore units and those with square pore units. regarding mechanical performance, Gong et al. [38] conducted fatigue resistance tests on triangular versus circular pore unit scaffolds, discovering that circular pore scaffolds exhibited uniform stress distribution without stress concentration, resulting in outstanding fatigue resistance.

### 2.2. Material Compositional Gradients

At the macroscopic scale, the transition of skeletal structure from peripheral cortical bone to internal cancellous bone [25] is characterized not only by structural variations in porosity and pore size but also by gradients in matrix composition density. While the primary constituents of the bone matrix consistently remain Type I collagen fibers and hydroxyapatite crystals, the degree of mineralization exhibits significant spatial variation. Cortical bone regions possess high mineralization, with collagen fibers aligned in a dense and ordered manner to withstand substantial axial loads; in contrast, cancellous bone regions exhibit relatively lower mineralization but higher metabolic activity, being rich in bone marrow mesenchymal stem cells (BMSCs) and vascular networks [39]. This implies that in the fabrication of bone scaffolds, it is necessary to introduce variations in matrix materials to mimic the mineralization and metabolic microenvironment of native bone.

Matrix materials employed for scaffold fabrication are primarily based on polymeric systems, which can be categorized into natural polymers, synthetic polymers, and composite polymers [40,41]. Natural polymers (e.g., collagen, gelatin, and hyaluronic acid) exhibit excellent biocompatibility due to their inherent bioactive constituents, enabling them to support cell adhesion, proliferation, and differentiation [42,43,44]. In contrast, synthetic polymers (e.g., polycaprolactone, polylactic acid, and polyetheretherketone) are typically biologically inert but often possess robust mechanical properties. To circumvent the limitations of single-material systems, composite polymer systems have emerged [40,43,45]. Composite polymers (e.g., GelMA, sodium alginate-hyaluronic acid, and dECM/alginate) combine the advantages of both natural and synthetic polymers; by incorporating functional components or multiphase structures, they simultaneously improve biological performance and enhance mechanical properties and processability, thereby better mimicking the complex microenvironment of the native extracellular matrix (ECM) [41]. Additionally, inorganic non-metallic materials such as hydroxyapatite and β-tricalcium phosphate exhibit excellent biocompatibility and osteoconductivity. They can mimic the inorganic composition of natural bone, providing an ideal three-dimensional growth space for bone cell adhesion, proliferation, and differentiation. These materials are also commonly used as bone scaffold materials [46,47].

The fundamental rationale behind constructing matrix material gradients lies in recapitulating the microenvironmental heterogeneity of natural bone tissue across the dual dimensions of chemical composition and biological signaling. Current strategies for constructing material compositional gradients primarily rely on dynamically modulating the blending ratios of natural and synthetic polymers [48] to alter the mechanical properties of the scaffold material, thereby achieving regulation of the scaffold’s mechanical strength. Concurrently, to cater to the regenerative demands of distinct tissue interfaces, the precise control over the spatial distribution of growth factors or functional peptides to establish specific biochemical concentration gradients has also emerged as a pivotal approach.

**Figure 2:**
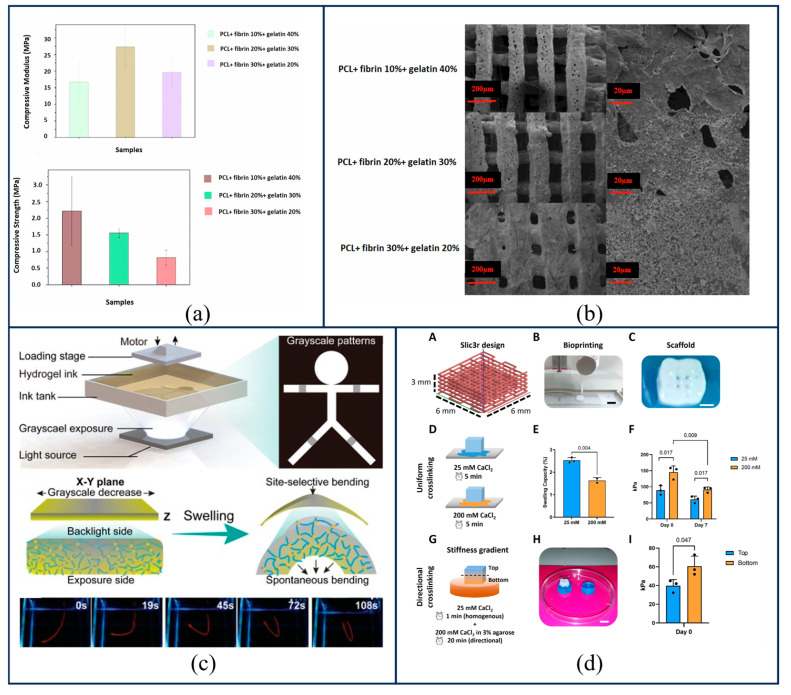
Material Composition Gradient and Crosslink Density Gradient: (**a**) Compressive strength and modulus of bilayer scaffolds with varying fibrin percentages, demonstrating the tunability of mechanical properties via composition control [50]; (**b**) Comparison of SEM images between as-fabricated bilayer scaffolds and those exhibiting surface degradation after 90 days of immersion in PBS [50]; (**c**) Fabrication of gradient hydrogels via grayscale stereolithography, where modulated light intensity dictates the local crosslinking density [52]; (**d**) Construction of stiffness-gradient scaffolds via directional ionic crosslinking, utilizing ion diffusion to establish a continuous mechanical gradient [53].

Construction strategies for material compositional gradients primarily encompass two main pathways: continuous gradient manufacturing and discrete stratified design. Regarding discrete gradient construction, researchers predominantly configure material systems with distinct chemical properties in different spatial domains to promote tissue-specific differentiation and interface integration. Li et al. [49] constructed an integrated bilayer 3D printed scaffold, achieving bidirectional induction of cartilage and bone through structural stratification and material coating; the upper layer of ECM/PCL stimulates chondrogenic differentiation, while the lower layer, containing agents such as MgO@PDA, promotes osteogenic differentiation. These studies demonstrate the feasibility of regulating scaffold performance by constructing material compositional gradients. Building on this, to further optimize mechanical matching, Maherani et al. [50] employed 3D printing technology to fabricate a bilayer scaffold for osteochondral tissue regeneration, comprising one layer of PCL/hydroxyapatite (HA) nanoparticles and another layer of PCL/gelatin containing varying concentrations of fibrin (10 wt%, 20 wt%, and 30 wt%). The compressive strength and compressive modulus of the bilayer scaffolds with different fibrin percentages are shown in Figure 2a, and a comparison between the SEM images of the as-fabricated bilayer scaffolds and those after surface degradation following 90 days of immersion in PBS is presented in Figure 2b, proving that such scaffolds possess good biodegradability and appropriate in vitro compressive properties. In pursuit of a higher degree of biomimicry, Wang et al. [19] designed a highly biomimetic three-layer mineralized collagen scaffold, consisting of a pure collagen surface region, an intermediate region of collagen and hydroxyapatite, and a wood-reinforced bottom region integrated with collagen and hydroxyapatite. This scaffold exhibited excellent biocompatibility and bioactivity. However, discrete layered structures may still suffer from stress concentration issues at the interface. In contrast, continuous gradients can more perfectly recapitulate the smooth transition characteristics of natural bone tissue. To this end, Zhang et al. [51] constructed a controllable gradient mixing platform for inorganic/organic biphasic biomaterials, achieving precise feed control of the sodium alginate/gelatin composite organic material and the hydroxyapatite inorganic material. This enabled the uniform mixing of the biphasic materials and the extrusion of the composite, resulting in the fabrication of an inorganic/organic biphasic gradient biomimetic bone scaffold with a matrix material gradient.

### 2.3. Crosslinking Density Gradients

The geometric structural and material compositional gradients discussed above achieve gradient construction primarily by altering the scaffold architecture and the material constituents themselves. Beyond these approaches, leveraging the physicochemical sensitivity of the material matrix offers an alternative strategy to construct gradients within a single homogeneous scaffold or to fine-tune the properties of scaffolds already possessing geometric or compositional gradients. Currently, the predominant methods involve the regulation of photo-crosslinking intensity and ionic crosslinking density. The core principle of this strategy lies in modulating the scaffold’s mechanical properties by varying the photo-crosslinking intensity or ionic crosslinking density, without altering the underlying chemical composition of the material.

The construction of photo-crosslinking gradients primarily relies on the on-line modulation of photoinitiator concentration via microfluidic printheads, or on the control of exposure intensity and duration. This approach enables the fabrication of a mechanical environment within the scaffold that features a continuous transition from soft to hard. Jiang et al. [52] realized a strategy for the programmable shape deformation of hydrogels using grayscale stereolithography technology; the designed grayscale patterns could control the spatial gradient of the hydrogel’s crosslinking density, thereby inducing changes in physicochemical properties. The process of fabricating gradient hydrogels via grayscale stereolithography is illustrated in Figure 2c. This technology successfully produced hydrogels with complex mechanical gradients, demonstrating the substantial potential of photo-controlled crosslinking in mimicking soft-to-hard tissue interfaces.

Ionic crosslinking gradients are achieved by regulating polymer concentration or crosslinker concentration to construct matrix mechanical gradients. This method circumvents the chemical compositional interference associated with the use of different materials, simulating the mechanical heterogeneity of bone from the superficial to the deep layers solely through a physical gradient of crosslinking density. Research by Frost et al. [54] fabricated scaffolds with stiffness gradients by varying the density of the crosslinker, thereby validating the feasibility of controlling gradients via ionic crosslinking. Building upon this, Hosseini et al. [53] employed a novel yet simple directional ionic crosslinking technique to fabricate biological scaffolds with stiffness gradients using a single type and concentration of biomaterial, as shown in Figure 2d. This strategy of constructing physical gradients within a single matrix offers significant insights for the future design of gradient scaffolds.

## 3. Design of Biomimetic Gradient Bone Scaffolds

### 3.1. Design Principles for Biomimetic Gradient Bone Scaffolds

The goal of bone tissue engineering is to restore and substitute the functions of bone tissue by integrating multidisciplinary technologies, including cell biology, biomaterials, and fabrication methods [29]. The core philosophy of hierarchical gradient design lies in overcoming the performance bottlenecks of traditional homogeneous materials or monolithic structures; this is achieved through the continuous or graded modulation of material composition, architecture, and properties across multiple dimensions to better recapitulate the structural characteristics of native bone tissue. As a pivotal element in bone tissue engineering, scaffolds must possess the following criteria to satisfy these requirements.

(1) Mechanical Adaptability: The modulus of the scaffold material should exhibit a spatial gradient to match the mechanical properties of different regions of the host bone [5]. The outer layer should possess a high modulus to match cortical bone, thereby preventing stress shielding; the inner layer should possess a low modulus and high toughness to match cancellous bone, facilitating stress transduction and stimulation.
(2) Biocompatibility: Bone scaffolds must possess excellent biocompatibility to provide adequate space for normal cellular activities such as adhesion, proliferation, and migration, while also ensuring the transport of nutrients. Upon implantation into the human body, the scaffold should not elicit immune rejection reactions [29].
(3) Bioactive Regionalization: By leveraging the intrinsic biochemical properties of the materials, microenvironments suitable for the maintenance of specific cell phenotypes or their differentiation should be constructed within different regions of the scaffold [24].
(4) Adequate Permeability: Permeability is critical for the transport of nutrients, the elimination of metabolic wastes, and vascular ingrowth; therefore, bone scaffolds must possess appropriate permeability [42].
(5) Biodegradability: Scaffolds should be biodegradable; furthermore, their degradation byproducts must be non-toxic and capable of being excreted from the body without interfering with the function of other organs [55].

### 3.2. Design Strategies for Biomimetic Gradient Bone Scaffolds

#### 3.2.1. Geometric Structural Gradient Design

As a pivotal element of bone tissue engineering, porous scaffolds and their structural design theories have consistently remained a primary focus and hotsp. Currently, the primary methodologies for designing the pore architecture of tissue engineering scaffolds include the unit cell array method, the Triply Periodic Minimal Surface (TPMS) method, medical image reverse engineering, and topology optimization. Scaffolds designed based on these differing methodologies exhibit distinct variations in performance.

(1) Unit Cell Array Method

The fundamental principle of the unit cell array method involves first defining a basic unit with simple geometry, referred to as the “unit cell.” Subsequently, utilizing Computer-Aided Design (CAD) technology, this unit cell undergoes periodic repetition and Boolean operations within three-dimensional space, thereby generating a porous scaffold model with a regular macrostructure. Chua et al. [56,57] established a library of common standard geometric models based on unit cells, which laid the foundation for this methodology. Researchers need only retrieve a unit cell model from the library and adjust its geometric parameters to efficiently construct a scaffold meeting specific porosity requirements. However, while this design strategy based on a standardized database significantly streamlines the process and enhances design efficiency, it remains challenging for this method to accommodate the construction of complex gradient structures.

(2) Triply Periodic Minimal Surfaces (TPMS)

**Figure 3:**
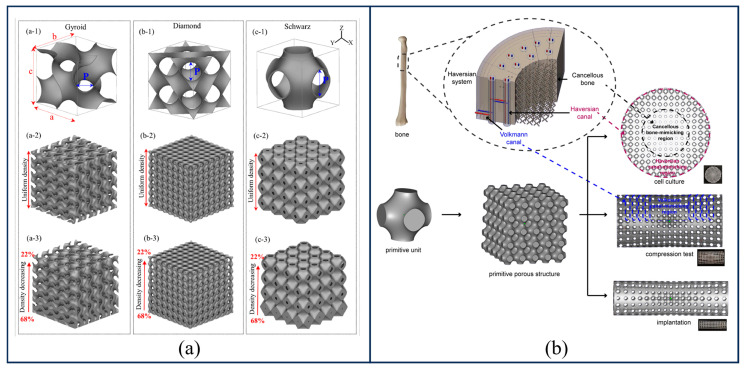
Design strategies for geometric structural gradients. (**a**) Construction of porous scaffolds using the Triply Periodic Minimal Surface (TPMS) method, allowing for precise control over pore geometry and permeability via topological variations [59]; (**b**) Schematic of the construction process for a Haversian system structure [60].

Triply Periodic Minimal Surfaces (TPMS) are a class of special surfaces strictly defined by mathematical implicit functions, which possess periodicity in three independent directions in three-dimensional space and have a zero mean curvature [58]. Common TPMS topologies primarily include the Primitive (P), Diamond (D), and Gyroid (G) types; the method for constructing scaffolds using these is illustrated in Figure 3a [59]. Compared to traditional truss-based structures, TPMS structures exhibit superior biomimetic and mechanical advantages. TPMS structures possess a fully interconnected pore network and an extremely high specific surface area, which not only significantly promote initial cell adhesion and proliferation but also provide efficient fluid channels for the transport of nutrients and metabolic wastes. Their surfaces are continuous and smooth, devoid of sharp geometric inflection points or nodes; this characteristic eliminates the stress concentration commonly found in traditional scaffolds and more closely approximates the native microenvironment within the body.

Researchers can precisely modulate the scaffold’s porosity, pore size, and mechanical modulus by adjusting the threshold constant (C value) or periodicity parameters within the implicit construction function, thereby achieving a precise match with host bone tissue characteristics. Precisely due to these unique advantages, the TPMS method has found extensive application in the design of tissue engineering scaffolds. In early research, Rajagopalan S et al. [61] designed a simple P-type surface scaffold based on the TPMS method, establishing the potential of this application in tissue engineering. Subsequently, Feng et al. [62] fabricated TPMS porous scaffolds using photocuring technology, demonstrating the feasibility of implicit surface porous scaffold design methods based on trigonometric functions. With the development of tissue engineering and the increasing complexity of scaffolds, numerous researchers have begun to explore advanced structural optimization and fusion strategies based on the TPMS method, proposing more refined design methodologies. Diez-Escudero A et al. [63] applied complex unit structures, such as Platonic and Archimedean polyhedral topologies, to scaffold design by computationally iterating shapes that most closely approximate the real structure of human bone. Yang et al. [64] proposed methods using Sigmoid functions and Gaussian Radial Basis Functions to pairwise fuse TPMS units, thereby obtaining hybrid structures.

(3) Medical Image Reverse Engineering

Medical image reverse engineering utilizes medical imaging data, such as Computed Tomography (CT) and Magnetic Resonance Imaging (MRI), as input. Through a series of digital processing steps performed by image processing software, the method reverse-engineers and reconstructs a three-dimensional model that matches the patient’s defect site with high precision. Distinct from the aforementioned forward design methods, the primary advantage of medical image reverse engineering lies in its ability to perfectly address the repair requirements of bone defects characterized by complex curved surfaces or irregular geometries.

Addressing the highly irregular geometric morphology of the human spine, Hille et al. [65] proposed a comprehensive reverse reconstruction workflow based on CT data. This work successfully reconstructed a vertebral model containing complex anatomical details, thereby demonstrating the application potential of this method. Furthermore, Hollister et al. [66] designed specific craniofacial biological scaffolds directly based on CT and MRI data. Collectively, these studies validate the feasibility of applying medical image reverse engineering within the field of bone tissue engineering scaffolds.

(4) Topology Optimization

The core principle of topology optimization involves utilizing mathematical algorithms to identify the optimal spatial distribution of materials within a given design domain. This process aims to maximize specific physical properties of the scaffold (such as stiffness and permeability) under defined boundary conditions, including porosity and manufacturing constraints. Design methodologies based on topology optimization enable precise control over pore structures, allowing the constructed porous models to satisfy design requirements across various application fields. Currently, mainstream topology optimization algorithms are widely applied in scaffold design. Shi et al. [67] optimized the structure of porous scaffolds using the Relative Density Mapping (RDM) method based on Finite Element Analysis (FEA) results, achieving superior load-bearing capacity compared to other structures at the same porosity. Guest et al. [68] applied the Solid Isotropic Material with Penalization (SIMP) method to optimize porous scaffolds, targeting elastic modulus and permeability as optimization objectives to realize a comprehensive optimization of mechanical and transport properties. Wu et al. [69] developed a scaffold design method based on the level-set topology optimization algorithm and time-dependent shape derivatives. Compared to typical scaffold structures, the optimized structures demonstrated significant advantages in sustained bone ingrowth. Huang et al. [70] achieved scaffold topology optimization based on the Evolutionary Structural Optimization (ESO) method (also known as the progressive structural optimization), obtaining maximum bulk modulus or shear modulus at different volume fractions. Li et al. [71] designed scaffolds with radial gradient pore structures based on topology optimization These studies collectively demonstrate the significant potential of topology optimization in the design of biomimetic gradient scaffolds.

Furthermore, to further enhance scaffold functionality from a biomimetic perspective, researchers have extended the design dimension to the microstructural level. Compared to the aforementioned gradient variations in macroscopic parameters, the topology optimization design of microstructures offers higher-resolution gradient regulation, thereby achieving the precise recapitulation of the complex hierarchical structure of native bone tissue. On one hand, Li et al. [60] utilized the TPMS method to design a gradient porous scaffold mimicking the Haversian system; the construction workflow is illustrated in Figure 3b. This design not only highly recapitulated the morphology of bone tissue but also achieved a profound alignment with native bone in terms of fluid transport efficiency and mechanical anisotropy. On the other hand, Xiao et al. [72], inspired by the Bouligand structure, proposed a microstructural regulation strategy based on printing path planning. By altering the filament angle in adjacent layers (ranging from 30° to 90°) and the filament spacing during the printing process (ranging from 0.8 mm to 2.4 mm), the mechanical properties of these modular samples could be modulated.

#### 3.2.2. Material Compositional Gradient Design

The spatial distribution of material composition determines the local physicochemical properties (such as stiffness, hydrophilicity, and degradation rate) and the biological response of the scaffold. Based on current research, the design of biochemical compositional gradients is generally categorized into two types: discrete gradients and continuous gradients. The patterns of gradient variation can be classified into linear, radial, exponential, orthogonal, and sigmoidal distributions [73]. Discrete gradient structures represent a solution with relatively simple manufacturing processes [50,74]. They involve switching print nozzles between different layers or regions to splice distinct areas with varying compositions, thereby constructing axial or radial gradients. However, this discontinuous construction approach results in distinct material interfaces between regions, subsequently inducing abrupt transitions in material properties between layers. Research indicates that such abrupt property changes lead to significant acoustic impedance mismatch and stress concentration; under physiological loading, once the peak shear stress at the interface exceeds the interlayer bonding strength, the implant becomes highly susceptible to fracture or delamination failure at the interface [75]. In contrast, continuous gradient structures employ a more optimized biomimetic strategy [12]. By enabling the volume fraction of constituents to vary continuously with spatial position, this approach completely eliminates distinct macroscopic interfaces. This architecture not only mimics the transitional characteristics of native tissues, such as the calcified cartilage layer, but more importantly, it effectively disperses interfacial stress over a volumetric region rather than concentrating it on a single plane, thereby avoiding stress concentration. Despite the superior performance of continuous gradients in addressing interfacial failure, this comes at the cost of significantly increased manufacturing complexity, necessitating reliance on high-precision dynamic mixing technologies and complex control algorithms for realization.

**Figure 4:**
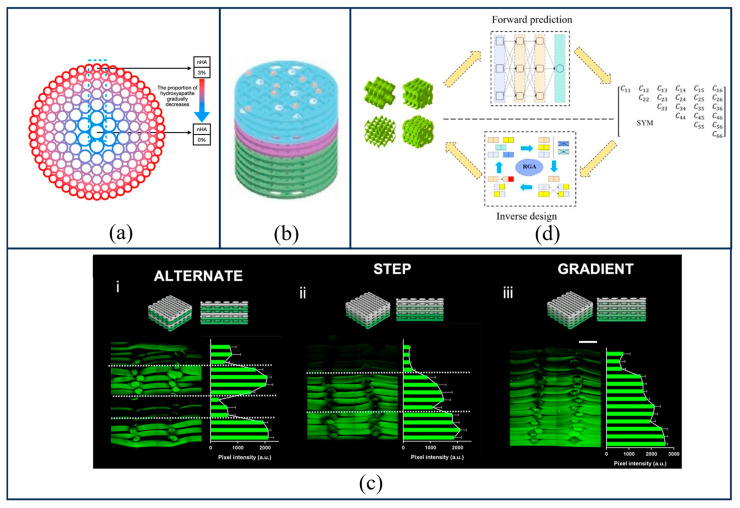
Strategies for multi-gradient bone scaffold design and optimization. (**a**) Schematic of a forward radial gradient structure, mimicking the anatomical transition from dense cortical shell to porous cancellous core [76]; (**b**) Schematic of a discrete axial gradient, composed of distinct layers with step-wise property variations [79]; (**c**) Schematic of a continuous axial gradient, engineered to ensure a seamless transition of properties and minimize interface stress concentrations [81]; (**d**) Workflow of forward prediction and inverse design using a Backpropagation Neural Network (BPNN), enabling the optimization of scaffold parameters for targeted mechanical and biological performance [82].

The spatial distribution strategies for biochemical compositional gradient design can be primarily categorized into radial gradients, axial gradients, and biomimetic microstructural gradients. The primary objective of designing radial gradients is to mimic the “hard-outer, soft-inner” architecture of native bone tissue. Current design strategies are divided into forward gradients and reverse gradients. A forward gradient [76], as shown in Figure 4a, employs high-strength material components (such as high-molecular-weight PCL or high-content HA/PCL composites) in the peripheral region to simulate the mechanical support function of cortical bone; meanwhile, the central region utilizes components with low modulus and high bioactivity (such as pure collagen, low-concentration PCL, or growth factor-doped hydrogels) to simulate cancellous bone and facilitate vascular ingrowth. Conversely, a reverse gradient [77], opposes the forward configuration. This strategy is primarily employed for specific applications (where degradation rates need to be tailored to bone growth), using slow-degrading materials with high HA content in the central region as a long-term core support, while the periphery comprises fast-degrading materials rich in TCP (tricalcium phosphate). This allows the outer layer to degrade rapidly to create space for nascent bone, while the center remains stable. Furthermore, research by Lee et al. [78] demonstrates that the presence of a radial gradient enhances the scaffold’s compressive and flexural properties, effectively mitigating stress shielding and stress concentration effects. Axial gradients primarily involve the repair of distinct tissue interfaces, as shown in Figure 4b [79]. The design strategy mimics the gradient transitions of bone-cartilage tissues and can be realized by switching materials or adjusting mixing ratios across different layers. Yang et al. [80] designed a biodegradable bilayer scaffold comprising a chondroitin sulfate (CS) hydrogel for regenerating cartilage tissue and a porous pure zinc (Zn) scaffold for regenerating the underlying bone and acting as mechanical support for the cartilage layer; this scaffold was proven to possess good cytocompatibility. Additionally, Serpe et al. [81] achieved a biological scaffold with continuous gradient variations by integrating microfluidics and a 3D bioprinting platform into a deposition system capable of providing distinct compartments with varying densities of cells and biomaterials, as depicted in Figure 4c.

### 3.3. Synergistic Multi-Gradient Design Methods

Regarding the design of biomimetic bone scaffolds, single structural gradients are often constrained by the performance bottlenecks of the matrix material itself, whereas single material gradients are prone to delamination failure due to insufficient interfacial bonding strength. In contrast, multi-gradient coupling strategies effectively integrate the dual advantages of geometric architecture and material composition: utilizing the geometric structure to enhance the bonding stability of material interfaces, while simultaneously leveraging the spatial distribution of materials to optimize the mechanical response of the structure. Such multi-gradient scaffolds achieve a more precise simulation of the complex transitions within native bone tissue and at interfaces by simultaneously modulating porosity and material composition along a specific direction, thereby realizing dual synergistic regulation of mechanical properties and cellular biological behaviors [83]. From a design perspective, an ideal bone scaffold must strike a balance between bioactivity and mechanical strength, which often represents a trade-off in practical design: while high porosity facilitates cell infiltration, nutrient transport, and bone regeneration, it significantly diminishes the scaffold’s mechanical support capability. Balancing this inherent conflict within multi-gradient structures constitutes the core problem that subsequent synergistic design methods aim to address.

A pivotal approach in synergistic multi-gradient design is the voxel-based design method. A voxel represents the smallest volumetric unit in three-dimensional space. Voxel-based methods discretize the scaffold into millions of such units, where each voxel can be independently assigned specific material properties and structural features, thereby achieving precise point-wise control over both structure and material at the microscopic level. Utilizing a voxel-based approach, Luo et al. [84] designed composite scaffolds composed of hydroxyapatite (HA) and polylactic acid (PLA). The process initially involves selecting appropriate porosity, pore shapes, and sizes to satisfy primary biological requirements; subsequently, the stiffness requirements are met by selecting suitable materials and adjusting their content. This methodology allows for the maintenance of high strength through the reinforcement of material properties while simultaneously preserving high porosity, thereby achieving a dual optimization of biological and mechanical performance within a single scaffold.

Implicit modeling technology offers an ideal mathematical framework for the synergistic optimization of structural topology and material composition. In this synergistic design strategy, the physical properties of the scaffold are collectively defined by two spatially coupled, yet independent, function fields. The structural gradient field governs the continuous evolution of geometric topology. By transforming the level-set threshold constant C in the Triply Periodic Minimal Surface (TPMS) equation into a spatial coordinate function C(x, y, z), designers can precisely modulate the gradient distribution of internal porosity and pore size [85]. The compositional gradient field defines the spatial distribution logic of heterogeneous materials, allowing designers to superimpose an independent material distribution function M(x, y, z) within the same geometric skeleton. This function dictates the mixing ratio of different material phases at specific spatial points [86]. This field-based dual mapping mechanism successfully overcomes the limitations of traditional designs where structure and composition are difficult to decouple.

In recent years, with the rapid advancement of Artificial Intelligence (AI), this technology has been increasingly applied to scaffold design. When strong non-linear conflicts exist between design objectives (e.g., permeability versus mechanical strength), employing multi-objective genetic algorithms (such as NSGA-II) in conjunction with surrogate models serves as an effective approach for identifying the Pareto frontier [87]. By constructing a comprehensive database containing tens of thousands of distinct TPMS unit cells alongside their corresponding Finite Element Analysis (FEA) and Computational Fluid Dynamics (CFD) simulation results, a Convolutional Neural Network (CNN) can be trained to serve as a predictive model [88]. During the design phase, this model can instantaneously predict the stiffness matrix for any given combination of design parameters, thereby substituting time-consuming FEA and CFD simulations. Subsequently, surrogate models (such as Kriging or Radial Basis Functions [RBF]) are employed to fit the response surface relating design variables to performance indicators. The algorithm then performs population evolution upon this response surface to rapidly screen thousands of design schemes, ultimately providing designers with a set of optimal trade-off solutions. Liu et al. [82] utilized a Backpropagation Neural Network (BPNN) to establish the mapping relationship between structural parameters and mechanical properties. Furthermore, a Regenerative Genetic Algorithm (RGA) was integrated with the machine learning model to perform an inverse search to obtain the desired structures, as illustrated in the workflow in Figure 4d.

## 4. Fabrication Processes of Biomimetic Gradient Bone Scaffolds

### 4.1. Fabrication Techniques for Biomimetic Gradient Bone Scaffolds

#### 4.1.1. Three-Dimensional Bioprinting Technology

Additive Manufacturing (AM) is a technology that constructs three-dimensional solid objects by accumulating materials layer by layer [89]. With the increasing maturity of 3D printing technology and the urgent clinical demand for tissue engineering scaffolds, 3D printing has been gradually applied to the field of biological tissue engineering fabrication. Among common additive manufacturing methods, 3D bioprinting, based on the principles of additive manufacturing, is a technology that formulates biomaterials, growth factors, and active cellular components into bio-inks, and fabricates three-dimensional structures with biological functions through layer-by-layer printing [90]. Compared with traditional 3D printing technology, 3D bioprinting faces additional complexities due to the introduction of bioactive components; this involves multiple factors such as material printability, diversity of cell types, and gradient structures. More importantly, the entire process must address technical challenges including maintaining high cell viability, matching mechanical properties with biological tissues, and ensuring superior biological performance [91,92]. Addressing these challenges requires the integration of technologies from multiple interdisciplinary fields, including engineering, biomaterials science, cell biology, physics, and medicine.

**Figure 5:**
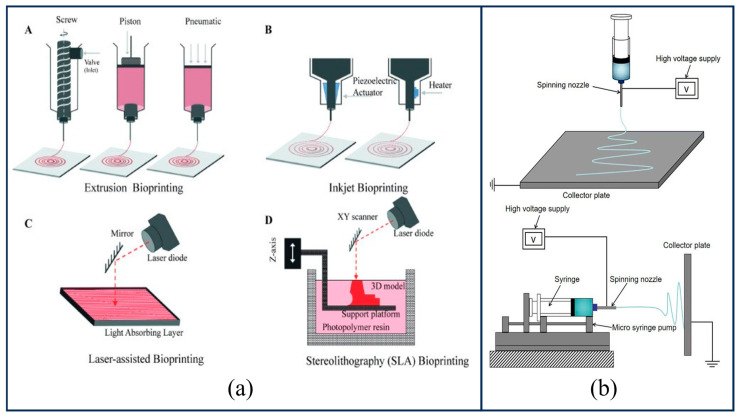
Additive Manufacturing Technology: (**a**) Schematic illustrations of the working principles of inkjet, micro-extrusion, and laser-assisted printing [93]; (**b**) Schematic diagram of the electrospinning system setup [99].

Based on the deposition and formation mechanisms of bio-inks during the printing process, current mainstream bioprinting technologies can be primarily categorized into inkjet bioprinting, micro-extrusion bioprinting, and laser-assisted bioprinting [91,93], with their operating principles illustrated in Figure 5a. Inkjet bioprinting operates by generating pressure pulses via thermal heating, piezoelectric, or acoustic actuation within the print head, thereby forcing droplets to eject from the nozzle [94,95]. Although studies have demonstrated that such thermal actuation does not compromise the stability of biomolecules [96], the operational mode of exposing cells and materials to thermal and mechanical stresses, combined with issues such as frequent nozzle clogging, severely restricts the broader application of this technology in bioprinting scenarios. Micro-extrusion bioprinting functions by applying controllable pressure—via a piston, screw, or pneumatic system—to the bio-ink within the nozzle [97]. This process extrudes the material through a nozzle to form continuous filaments, which are deposited layer-by-layer onto a platform to construct a three-dimensional structure. This method is particularly well-suited for non-Newtonian fluids exhibiting shear-thinning properties. During the extrusion process, the material’s viscosity decreases under shear stress, facilitating flow through the nozzle; upon deposition, the removal of shear stress allows the viscosity to recover, thereby ensuring excellent structural stability. Laser-assisted bioprinting utilizes focused laser pulses to induce the generation of transient high-pressure bubbles within an energy-absorbing layer [98]. This drives the bio-ink to form a micro-jet that is precisely deposited onto a receiving substrate, thereby achieving high-resolution, nozzle-free, and non-contact biofabrication. While this nozzle-free design achieves single-cell level precision and high cell viability, its ability to rapidly construct large-scale three-dimensional tissues is limited by high equipment costs and complex preparation protocols. Among the aforementioned bioprinting technologies, extrusion-based 3D printing has found widespread application in the field of biofabrication and bioprinting, primarily because its fabrication environment is highly benign to bioactive substances such as living cells and growth factors.

#### 4.1.2. Electrospinning Technology

Electrospinning technology is a micro/nano-fabrication method based on electrohydrodynamic (EHD) principles. The system setup, as illustrated in Figure 5b, primarily consists of a spinneret (or ejector system), a collector, and a high-voltage power supply. Under the influence of a high-voltage electric field, polymer droplets deform into a Taylor cone and are subsequently stretched into a jet; following solvent evaporation, the material deposits onto the receiving device to form fibrous membranes or three-dimensional structures [99]. This process has emerged as a crucial strategy for bone scaffold fabrication, primarily due to its ability to highly mimic the topological structure and fibrous scale of the natural extracellular matrix (ECM). It can effortlessly fabricate fibers with diameters at the micrometer scale, providing a favorable microenvironment for cell adhesion, proliferation, migration, and differentiation [100]. The resulting electrospun nanofibers possess an extremely high specific surface area and an interconnected porous network. This architecture not only provides ample space for cellular activities but also facilitates the transport of nutrients and the removal of metabolic wastes. Furthermore, by modulating solution properties, process parameters, or the receiving assembly, the fiber orientation, stacking pattern, and pore structure can be controlled to a certain extent, thereby meeting the specific requirements for the directional regeneration of bone tissue [101].

However, traditional electrospinning technology also possesses distinct limitations when applied to tissue engineering scaffolds. Residual organic solvents may induce cytotoxicity and hinder the loading and activity retention of bioactive factors [102]. Furthermore, severe whipping instability exists during the printing process, resulting in stochastic fiber deposition and making it difficult to precisely control the spatial arrangement of individual fibers. Nanofiber membranes formed by the dense packing of disordered fibers often exhibit limited porosity, which may impede deep cellular infiltration into the scaffold and vascular ingrowth [103], posing a significant challenge for the repair of critical-sized bone defects. To overcome the deficiencies of traditional electrospinning in control precision, Near-Field Electrospinning (NFES) technology has emerged. By shortening the distance between the spinneret and the collector and reducing the applied voltage, the whipping instability of the jet is suppressed. This allows the jet to deposit along a nearly straight trajectory, constructing scaffolds with defined geometric patterns, controllable pore structures, and complex three-dimensional morphologies [104]. Compared to traditional electrospinning, the lower voltage conditions during NFES reduce potential damage to sensitive bioactive molecules. Moreover, scaffolds constructed via this direct-writing manner typically feature larger and more regular pore structures, facilitating cell infiltration, vascularization, and nutrient transport. NFES provides new avenues for multi-material and multi-functional integrated manufacturing. For instance, it allows for the alternating direct writing of fibers from different materials to construct mechanical or chemical gradient scaffolds, or can be hybridized with other bioprinting technologies to jointly fabricate more complex biomimetic bone tissue structures.

#### 4.1.3. Physicochemical Preparation Techniques

Despite the rapid advancement of additive manufacturing technologies, traditional physicochemical preparation methods retain a significant role in the fabrication of bone scaffolds. This is attributed to their low equipment requirements, cost-effectiveness, suitability for large-scale production, and specific advantages in processing certain materials. The primary preparation techniques include freeze-drying, solvent casting, thermally induced phase separation (TIPS), and gas foaming.

(1) Freeze-drying

**Figure 6:**
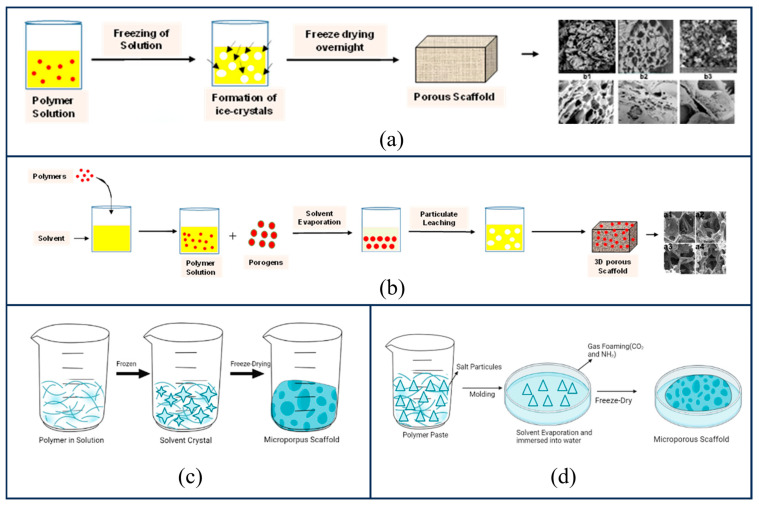
Physicochemical strategies for fabricating porous scaffolds. Schematic diagrams and microscopic images of the freeze-drying (**a**) and solvent casting (**b**) processes [106]; schematic diagrams of the thermally induced phase separation (**c**) and gas foaming (**d**) processes [46].

Freeze-drying is particularly well-suited for heat-sensitive natural polymers such as collagen and gelatin. Its preparation workflow is illustrated in Figure 6a. The fundamental principle involves freezing the polymer solution to induce solvent crystallization (typically water forming ice crystals), followed by removing the solvent via sublimation under vacuum conditions; the spaces previously occupied by the ice crystals subsequently evolve into the scaffold’s pores [105]. The morphology of the pores is dictated by the growth of the ice crystals. By controlling the freezing temperature gradient, ice crystals can be guided to grow along a specific direction, resulting in the formation of aligned microtubular channels after sublimation. Such oriented structural scaffolds are capable of guiding cell migration as well as the directional ingrowth of nerves and blood vessels. However, scaffolds prepared by this method often suffer from issues such as small pore sizes and irregular porosity, which severely restrict their widespread promotion and application [106].

(2) Solvent Casting

The Solvent Casting method employs porogens to regulate the pore structure [107]. The fabrication workflow, illustrated in Figure 6b, involves dissolving the polymer in an organic solvent and incorporating insoluble porogen particles (such as sodium chloride salt particles, sugar spheres, or paraffin spheres). Following solvent evaporation, the polymer solidifies, encapsulating the porogens. Finally, water or another specific solvent is utilized to dissolve and leach out the porogens, thereby leaving behind a porous architecture [106]. The pore size is determined by the dimensions of the porogen particles, while the porosity is governed by the quantity of porogen added; this endows the pore structure with superior controllability. The primary drawbacks of scaffolds prepared by this method lie in the limited film thickness, relatively poor pore connectivity, and the potential cytotoxicity of residual organic solvents.

(3) Thermally Induced Phase Separation (TIPS)

Thermally Induced Phase Separation (TIPS) utilizes the thermodynamic instability of polymer solutions to prepare porous scaffolds. By adjusting the polymer concentration, quenching temperature, and solvent system, TIPS can fabricate a variety of microstructures; the process flow is illustrated in Figure 6c. The advantage of this technology lies in its ability to permit the incorporation of bioactive agents and its compatibility with various other manufacturing techniques to design three-dimensional structures with controlled pore morphology [108]. Due to its capacity to mimic the native extracellular matrix (ECM) and its controllable pore size, it has been widely applied across various fields [46]. Research by Conoscenti et al. [109], based on the TIPS method, constructed a polylactic acid (PLA) scaffold featuring a continuous axial pore size gradient, ranging from a diameter of 70 μm on the cartilage repair side to over 200 μm on the bone repair side. It was found that this scaffold could provide unique and independent signaling cues for cartilage and bone differentiation, while simultaneously permitting communication across the osteochondral junction, mimicking the in vivo environment.

(4) Gas Foaming

The preparation workflow of the gas foaming method is illustrated in Figure 6d [46,110]. This technique utilizes high-pressure gas (such as carbon dioxide or nitrogen) as a porogen. During the polymerization or processing stage, an increase in temperature or a reduction in pressure causes the gas to escape, thereby generating porosity. Scaffolds fabricated via this method can achieve porosity levels reaching 85–93% [108]. Furthermore, this approach avoids the use of organic solvents, making it particularly suitable for loading bioactive substances such as growth factors.

#### 4.1.4. Hybrid Manufacturing Technology

Single manufacturing techniques often fail to simultaneously satisfy the comprehensive requirements of bone scaffolds regarding macroscopic mechanical strength, microscopic pore structure, and nanoscopic surface morphology. Consequently, hybrid manufacturing strategies have emerged, aiming to integrate the distinct advantages of diverse technologies [111]. The combination of 3D printing and electrospinning represents the most prominent hybrid strategy currently, synergizing the mechanical toughness of the 3D-printed framework with the bioactivity of the electrospun matrix. Although 3D printing technology is capable of constructing customized macroscopic scaffolds with interconnected pores to facilitate nutrient transport, it is constrained by printing resolution and thus struggles to simulate the intricate microstructures of native tissues. Specifically, the pore sizes produced by 3D printing are often significantly larger than the cellular scale; this discrepancy may lead to low cell seeding efficiency and impede tissue integration. In contrast, electrospinning technology enables the fabrication of nanofibers that structurally mimic the extracellular matrix (ECM) to a high degree. This microstructure not only provides abundant binding sites for cell attachment and proliferation but also serves as a carrier to achieve the controlled release of bioactive molecules, thereby more effectively supporting tissue regeneration [98].

**Figure 7:**
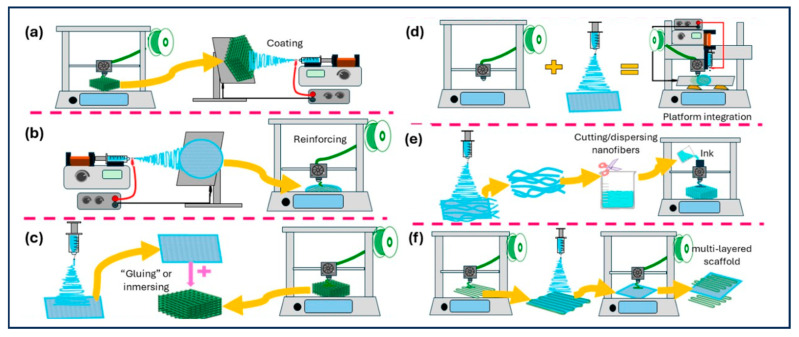
Fabrication processes and performance comparison of hybrid 3D printing and electrospinning strategies [112]. (**a**) Application of electrospinning technology to 3D printed scaffolds. (**b**) Application of 3D printing technology to electrospun fibers. (**c**) Decoration/impregnation of 3D printed scaffolds with electrospun nanofiber segments. (**d**) Platform combining 3D printing with electrospinning technology. (**e**) Electrospun fibers used as ink for 3D printing. (**f**) Alternate application of 3D printing and electrospinning technology.

Methods combining 3D printing and electrospinning are illustrated in Figure 7a. These strategies include electrospinning onto 3D-printed scaffolds, 3D printing onto electrospun fibers, modifying 3D-printed scaffolds with electrospun nanofiber segments, utilizing platforms that integrate both 3D printing and electrospinning technologies, employing electrospun fibers as inks for 3D printing, and the alternating application of 3D printing and electrospinning [112]. Wang et al. [113] fabricated scaffolds using a combined approach of 3D printing and electrospinning. Building upon this, Yu et al. [114] similarly utilized electrospinning and 3D printing technologies to manufacture 3D composite scaffolds. They conducted a comparative analysis of the mechanical properties and biological performance among pure electrospun scaffolds, pure 3D-printed scaffolds, and hybrid-manufactured scaffolds. The results presented demonstrate that the composite scaffolds not only exhibit superior mechanical properties and biocompatibility but also facilitate cell migration and proliferation. These findings indicate that electrospinning/3D printing composite scaffolds possess significant potential for the repair and regeneration of bone tissue.

### 4.2. Gradient Construction Methods for Biomimetic Bone Scaffolds

#### 4.2.1. Multi-Nozzle Extrusion

**Figure 8:**
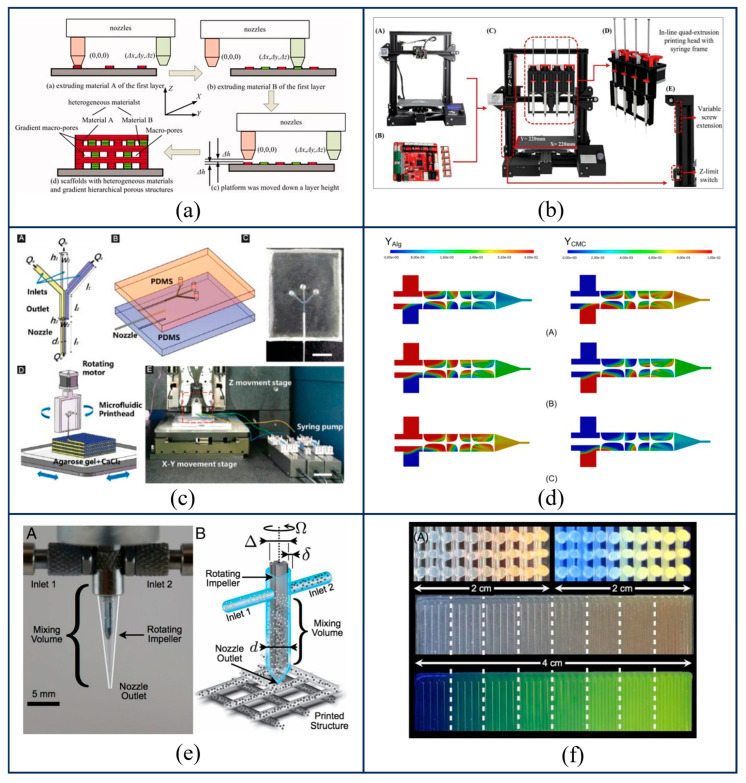
Advanced manufacturing strategies for composite gradient scaffolds. (**a**) Schematic of the multi-nozzle switching process, forming discrete gradients by alternating material deposition [115]; (**b**) Configuration of the multi-nozzle printing system equipped with independent extrusion heads [116]; (**c**) Schematic of the real-time mixing printing process, designed to blend inks in situ for continuous gradient generation [120]; (**d**) Computational fluid dynamics (CFD) simulation of a static mixer [121]; (**e**) Design of an active mixer nozzle incorporating dynamic stirring to enhance component uniformity [122]; (**f**) Cross-sectional view of a printed lattice, verifying the successful fabrication of a continuous composition gradient [122].

Multi-nozzle extrusion systems represent the most direct and established approach for constructing discrete gradient scaffolds. Such printers are typically equipped with two to four, or even more, independent cartridges and extrusion nozzles. Each cartridge is loaded with bio-inks or thermoplastic polymers of varying composition, concentration, or cell type. A typical application of this technology is the construction of soft-hard composite scaffolds. Utilizing slicing code (G-code) generated by Computer-Aided Design (CAD) software, the control system can automatically switch the active nozzle according to a preset path during the printing process, or direct different nozzles to deposit material within distinct regions, thereby achieving a gradient variation along a specific direction of the scaffold. The process of scaffold fabrication via multi-nozzle switching is illustrated in Figure 8a [115]. Multi-nozzle extrusion systems are the most straightforward means to achieve this objective. Zgeib et al. [116] developed a low-cost four-nozzle 3D bioprinting system for multi-material tissue construction, as shown in Figure 8b. This strategy excels in constructing multiphase tissues characterized by distinct interfaces. Yu et al. [24] utilized multi-nozzle bioprinting to prepare GelMA-based biphasic scaffolds, with the upper layer loaded with chondrocytes and the lower layer doped with Sr-CSH. This achieved a synergistic gradation of material composition and structure, successfully inducing zonal differentiation. Furthermore, addressing challenges such as the poor printability of pure collagen and the difficulty in maintaining structural integrity during multi-layer printing with multi-nozzle switching, Yao et al. [117] improved collagen printability by optimizing its concentration and pH values. They also accomplished the large-span printing of thermoplastic elastomers utilizing a precise temperature control system. Addressing the issues of delay and overflow (oozing) caused by the start and stop of nozzle switching, Liu et al. [118] developed a multi-channel bundled nozzle. Although physically remaining multi-channel, the nozzles converge at a proximate outlet; combined with rapid pneumatic switching, this realized quasi-continuous fiber deposition. To compare the differences between discrete and continuous gradients, Bedell et al. [119] compared the performance of scaffolds with abrupt interfaces versus gradual interfaces. They found that while the segmented design offered advantages in mineralization, the gradient structure significantly enhanced the integration with host tissue.

Despite the maturity and cost-effectiveness of multi-nozzle technology, its intrinsic limitations restrict the resolution of the generated gradients. At switching points or interfaces between layers, different materials (particularly hydrophobic polymers and hydrophilic hydrogels) often rely solely on physical contact, lacking chemical bonding; this results in the interface becoming a mechanical weak point [120,123]. The method of switching nozzles fails to recapitulate the smooth gradient transitions found in native tissues. The abrupt mutations in properties between layers may lead to discontinuities in the mechanical signals sensed by cells. From a processing standpoint, frequent nozzle start-stop operations are prone to inducing pressure hysteresis in non-Newtonian fluid inks at the nozzle tip, causing material accumulation or flow interruptions at the starting point of printing.

#### 4.2.2. Real-Time Mixer

To overcome the intrinsic limitations of the multi-nozzle switching method, the introduction of fluid mixers to enable the construction of continuous gradients has emerged as a significant research hotspot in recent years. This system typically comprises two or more high-precision syringe pumps connected to a shared mixing nozzle, as illustrated in Figure 8c [120]. By dynamically adjusting the flow rate ratios of the respective syringe pumps in real-time, the proportion of different ink components entering the mixing chamber varies continuously over time. The mixed material is subsequently extruded, thereby forming a continuous material compositional gradient within a single filament or throughout the entire scaffold structure [124]. Based on their working principles, these mixers can be categorized into static mixers and dynamic mixers.

Static mixers contain fixed geometric elements, such as helical blades or baffles, which force fluids to undergo repeated splitting, rotation, and recombination as they flow through [125]. Their mixing mechanism primarily relies on laminar division and molecular diffusion [126]. Hardin et al. [127] designed a multi-material 3D printing printhead based on microfluidic technology, capable of rapidly switching between multiple inks within a single nozzle. However, the mixing efficiency of this approach is highly dependent on the path length and the number of elements. It is particularly difficult to achieve homogeneous mixing when handling materials with significant viscosity mismatches or extreme flow rate ratios. Attempts to improve mixing quality by extending the mixing tube significantly increase the residence time of the fluid from inlet to outlet. This results in severe lag effects in gradient generation, making rapid gradient switching difficult to achieve [126,128]. To address these challenges, researchers have proposed various optimization strategies. Gharraei et al. [121] utilized Computational Fluid Dynamics (CFD) simulations to study the flow and mixing behavior of materials. Based on these behaviors and governing laws, they redesigned the bioprinting head. The simulation results of the mixing process, as shown in Figure 8d, indicate that the redesigned head achieved complete mixing of biomaterials. Furthermore, the transition time (defined as the delay between a change in inlet flow rate and the corresponding change in fiber composition at the outlet) could be effectively regulated or reduced by pre-adjusting the inlet flow. Similarly based on CFD methods, Wei et al. [129] investigated the influence of mixer structural parameters on fluid shear stress and mixing efficiency, providing an optimized design based on the analysis. Finally, hydrogel mixing printing experiments were conducted to validate the mixer’s performance. Additionally, Serex et al. [130] designed a printhead based on microfluidics composed of three microchannels that converge into one just before the ejection point. Each of these three channels can be connected to a syringe containing a different material and driven by a syringe pump. This setup allows for seamless transitions between multiple materials during object fabrication. Due to the extremely small dead volume, a complete transition between two materials can be achieved within 500 ms. These studies provide theoretical guidance for the design of mixing extrusion nozzles in 3D bioprinting.

To overcome the limitations of passive mixing, researchers have conducted studies on dynamic mixers (also known as active mixers). Dynamic mixers integrate rotating impellers (or blades) driven by micro-motors, as illustrated in Figure 8e [122]. These devices generate intense chaotic advection within a miniature mixing chamber, thereby circumventing the limitations associated with passive mixing [129,131]. In bioprinting applications, the high shear rates generated by the active stirring of dynamic mixers effectively exploit the shear-thinning behavior of non-Newtonian fluids such as bio-inks. This significantly reduces the apparent viscosity of high-viscosity materials, thereby narrowing the viscosity mismatch between heterogeneous materials. Consequently, rapid and homogeneous mixing can be achieved even at extremely low flow rates, effectively enhancing printing resolution. The cross-sectional image of a rectangular lattice structure printed by Ober et al. [122], shown in Figure 8f, clearly exhibits a material gradient, validating its mixing performance. Teves et al. [132] further developed a printhead capable of processing liquid bicomponent materials alongside a third component, thereby enabling rapid compositional transitions during the continuous extrusion process. Furthermore, owing to the extremely high mixing efficiency, the volume of the mixing chamber can be designed to be highly miniaturized. This drastically improves time resolution and eliminates gradient lag (hysteresis). However, it is noteworthy that in practical applications, the shear stress levels generated by rotation must be strictly controlled to prevent excessive mechanical forces from damaging the encapsulated living cells within the ink. To address this issue, Hidaka et al. [133] began exploring gentle mixing strategies, proposing a novel method that achieves uniform mixing of multiple inks in 3D bioprinting via acoustic stimulation, demonstrating gentle yet effective multi-ink mixing.

#### 4.2.3. Coaxial Printing/Spinning

Coaxial printing/spinning is an advanced micro/nano-fabrication technology capable of precisely constructing bone scaffolds with spatial gradients across micro- to macroscopic scales. Its core principle involves utilizing a specially designed concentric nozzle (or needle) system to co-extrude two or more fluids with distinct properties under the influence of an electric field (coaxial electrospinning) or mechanical pressure (coaxial 3D printing). Consequently, these fluids solidify to form fibers, particles, or tubular materials possessing well-defined core–shell or multi-layer architectures.

(1) Coaxial Electrospinning Technology

**Figure 9:**
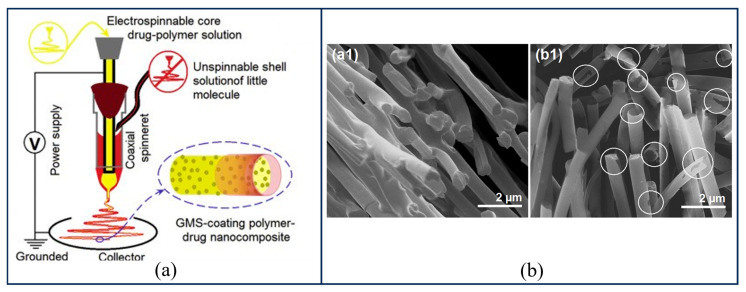
Coaxial Printing/Spinning: (**a**) Schematic diagram of coaxial electrospinning [135]; (**b**) SEM images of the nanofiber’s cross-sections and their estimated diameters [135].

The principle of coaxial electrospinning [134,135] involves modifying the spinneret structure based on traditional electrospinning, as illustrated in Figure 9a. By concentrically embedding a smaller capillary within a larger one to establish a coaxial configuration, both the inner and outer needles can simultaneously pump the core and shell solutions, thereby forming a core–shell structured droplet at the nozzle exit. This core–shell architecture overcomes the limitations inherent to traditional uniaxial electrospinning. In traditional uniaxial electrospinning, the direct exposure of bioactive molecules to organic solvents often results in protein denaturation and inactivation. Furthermore, drug molecules distributed on the fiber surface are highly prone to uncontrollable burst release effects, causing excessively high local concentrations. In contrast, the shell layer in the coaxial structure not only serves as a physical barrier that effectively isolates the core payload from the harsh external environment but also achieves zero-order release or sustained release of drugs by regulating the diffusion distance [136]. When a high voltage is applied, charges primarily accumulate on the surface of the outer shell solution. Under the influence of electrostatic repulsion, the pendant shell droplet is stretched and deformed into a conical shape (Taylor cone). Once charge accumulation reaches a threshold, a jet ejects from the droplet tip towards the counter electrode, ultimately depositing onto the substrate to form fibers with a core–shell structure. The stable formation of the shell-core structure and the fiber morphology in coaxial electrospinning mainly depend on the comprehensive regulation of solution parameters (e.g., viscosity, concentration, conductivity, solvent miscibility, and volatility) and processing conditions (e.g., voltage, flow rate, collection distance, and nozzle geometry) [134]. The shell solution typically requires higher viscosity and conductivity to generate sufficient traction force and refine the fibers, whereas the core solution requires lower viscosity and flow rate to maintain continuity [137]. Simultaneously, solvent selection must balance compatibility and evaporation rate to prevent structural collapse or nozzle clogging. The compatibility at the core–shell interface is a critical factor determining the mechanical properties of the fibers. Furthermore, an appropriate voltage range, the flow rate ratio between shell and core fluids (typically, the core flow rate is lower than the shell flow rate) [138], collection distance, and nozzle design are crucial for maintaining the stability of the Taylor cone, ensuring sufficient solvent evaporation, and obtaining uniform and smooth nanofibers.

In the field of bone tissue engineering, this technology endows scaffolds with the capabilities to achieve controlled release of drugs/growth factors, mimic the microstructure of the bone matrix, and induce vascularization. For instance, nanofibers prepared by Hai et al. [135] using the coaxial electrospinning method are shown in Figure 9. Wu et al. [139] utilized gelatin loaded with CT as the shell solution and PCL loaded with SLA as the core solution to fabricate nanofibrous membranes via coaxial electrospinning. They found that the nanofibrous membranes loaded with SAL and CT exhibited superiority in promoting angiogenesis and bone repair, while demonstrating excellent biocompatibility compared to other membranes. The coaxial structure of the nanofibrous membranes enabled long-term, spatially targeted drug release, thereby promoting bone repair and angiogenesis in rat bone defects. Similarly, He et al. [140] employed coaxial electrospinning to prepare nanofibers featuring an APR core layer and a TP shell layer. This achieved controlled drug release, capable of assisting the bone remodeling process in periodontitis, effectively controlling inflammation, and promoting bone regeneration.

(2) Coaxial 3D Printing Technology

Coaxial 3D printing operates on a principle similar to coaxial electrospinning. It utilizes nested concentric nozzles to introduce two bio-inks with distinct properties into independent inner and outer channels. By maintaining a stable interface at the extrusion orifice through rheological matching, and leveraging ionic permeation or photo-initiators to achieve rapid in situ crosslinking, this technology ultimately constructs continuous fibers or hollow tubular structures with distinct core–shell stratified features [141].

Coaxial nozzle technology allows the bio-ink and crosslinker to contact and crosslink in situ at the instant of extrusion, realizing a fast, controllable transition from a liquid precursor to a solid gel. This rapid gelation process immediately fixes the morphology of the extruded filament, effectively preventing spreading, collapse, or coalescence deformation during deposition. In ionic crosslinking systems, the most classic application is the sodium alginate and calcium chloride (CaCl_2_) system. Typically, sodium alginate and CaCl_2_ solutions serve as the shell and core layers of the coaxial nozzle, respectively. When the two solutions contact at the nozzle exit, calcium ions diffuse rapidly, triggering the gelation of the alginate and instantaneously forming hollow tubular or solid fibrous structures [142]. To ensure printing stability and reliability, Sun et al. [143] investigated theoretical models of coaxial printing and defined the stable processing window. Their results indicated that optimal scaffold characteristics are achieved when using a 3% sodium alginate solution paired with a 2.5% CaCl_2_ solution, ensuring the alginate flow rate is maintained at a minimum of 0.5 mL/min, with the CaCl_2_ flow rate being approximately double that of the alginate. In addition to ionic systems, for photosensitive materials such as GelMA (Gelatin Methacryloyl), coaxial printing can similarly leverage their crosslinking effects. O’Connell et al. [144] proposed an in situ photo-crosslinking strategy, which allows the bio-ink to crosslink rapidly (<1 s) upon extrusion from the nozzle. Furthermore, the coaxial printing method can mitigate cell damage during the printing process. in uniaxial 3D printing, cells passing directly through narrow printing nozzles are subjected to high shear stress, leading to decreased cell viability [145]. Coaxial 3D printing overcomes this issue by placing the cell-laden bio-ink in the core layer, encapsulated by structural material in the shell layer. Since the outer fluid bears the primary frictional shear against the nozzle wall, the mechanical damage to the cells in the core layer is significantly reduced. Blaeser et al. [145] confirmed via Computational Fluid Dynamics (CFD) simulations that the shear stress within the coaxial nozzle exhibits a parabolic distribution that decreases from the wall toward the center. This proves that scaffolds prepared via coaxial 3D technology contribute to enhanced cell survival [146].

#### 4.2.4. Gradient Photocuring

**Figure 10:**
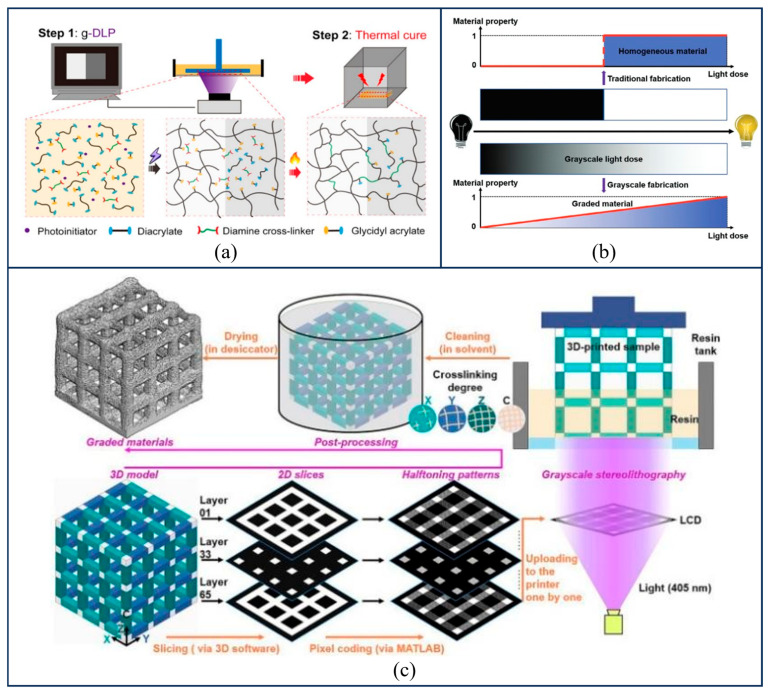
Strategies for light-induced gradient fabrication. (**a**) Schematic of gradient photopolymerization, utilizing spatially modulated light exposure to generate crosslinking density gradients [148]; (**b**) Comparison illustrating the binary nature (on/off) of conventional photopolymerization versus the continuous intensity control of grayscale photopolymerization [150]; (**c**) Schematic of halftone grayscale 3D printing.

Photocuring technology refers to the utilization of the property of photosensitive biomaterials (such as GelMA and PEGDA) to undergo polymerization crosslinking under irradiation at specific wavelengths, as illustrated in Figure 10a [147,148]. By precisely spatially regulating the light dosage (e.g., by altering light intensity or exposure time), different degrees of polymerization crosslinking are induced in the photosensitive material, thereby directly fabricating functional materials that exhibit gradient changes in mechanical properties [149].

Traditional Digital Light Processing (DLP) technology employs a Digital Micromirror Device (DMD) to project slice images of each layer onto the liquid resin surface. These images are typically binary, meaning the material within a region is either fully cured or remains uncured. Gray-scale DLP technology introduces gradient control of light intensity. Through software algorithms, slice images are converted into grayscale maps. The DMD then controls the flipping frequency of each micromirror via Pulse Width Modulation (PWM) based on the pixel’s gray value, thereby precisely regulating the light dosage projected onto every point on the resin [150]. The comparison between traditional and gray-scale photocuring is shown in Figure 10b. The degree of the photopolymerization reaction is positively correlated with the light dosage. Higher light intensity and longer exposure times generate more photoinitiator radicals, resulting in a higher crosslinking density of the polymer network. Since the mechanical modulus of polymer materials is typically positively correlated with crosslinking density, gray-scale modulation enables the construction of continuous gradients of mechanical properties within a single material, without the need to alter the material’s chemical composition. Fei et al. [151] developed a digital halftoning method to generate universal printable files consisting of black and white pixels. Using this method, any printer can control the irradiation dose and degree of photopolymerization for each printed layer; the workflow is shown in Figure 10c. Ding et al. [152], based on photocuring principles, created a tunable photo-crosslinking gradient by directly incorporating UV absorbers into photo-crosslinkable biopolymers. This gradient enabled the hydrogel to undergo on-demand, controllable shape morphing (or remodeling) while maintaining excellent cytocompatibility. Yin et al. [153] proposed a multi-wavelength stepwise curing strategy. By utilizing the wavelength-selective characteristics of light absorbers, this approach realized gradient regulation of the printed part’s mechanical properties while ensuring high biocompatibility. It addressed toxicity issues and simultaneously achieved multi-scale mechanical regulation for medical applications.

## 5. Conclusions and Future Perspectives

In summary, this review systematically elucidates the research progress of biomimetic bone tissue engineering scaffolds from three dimensions: gradient characteristic analysis, design strategy evolution, and manufacturing process implementation. First, regarding the understanding of gradient characteristics, this paper summarizes the key gradient attributes of natural bone tissue. This covers the geometric structural gradients of macroscopic pore distribution, the material compositional gradients of microscopic matrix variations, and the crosslinking density gradients that determine mechanical properties. A profound understanding of these multi-dimensional gradient features serves as the biological foundation for achieving biomimetic construction and functional regeneration. Second, in terms of scaffold design strategies, this paper not only proposes design requirements for gradient scaffolds and summarizes design philosophies for geometric and compositional gradients but also reviews representative achievements in multi-gradient integration. Early designs primarily focused on simulating single physical structures or biochemical components, whereas current research is progressively shifting toward multi-gradient synergistic design. By integrating physical structural gradients with biochemical compositional gradients, it is possible to more precisely simulate the complex in vivo microenvironment, providing optimized inductive conditions for cell adhesion, proliferation, and osteogenic/angiogenic differentiation. Finally, regarding manufacturing implementation, this paper focuses on analyzing the application potential of bioprinting, electrospinning, physicochemical methods, and their hybrid manufacturing technologies in gradient construction. In particular, the application of advanced techniques—such as multi-nozzle switching extrusion, real-time mixing systems, coaxial printing/spinning, and gradient photocuring—has achieved spatiotemporal regulation over the internal microstructure and component distribution of scaffolds. These technological breakthroughs have successfully translated complex multi-gradient design concepts into physical scaffolds, significantly enhancing the biomimetic level and clinical repair potential of the scaffolds. In conclusion, research on biomimetic gradient bone scaffolds has evolved from simple structural mimicry to a multi-gradient systematic engineering effort that integrates structure, composition, and function, providing a promising solution for the repair of complex bone defects.

Despite significant progress in mimicking the heterogeneous structure and function of natural bone, current research and development still rely primarily on empirical forward design. Furthermore, most scaffolds provide only passive physical support post-implantation, making it difficult to fully adapt to the complex spatiotemporal demands of the dynamic regeneration of bone tissue. Future research should focus on the deep integration of the following three frontier directions:

(1) AI-Aided Topology Optimization and Inverse Design: Traditional gradient designs are often based on simplified linear models, making it difficult to cope with the highly irregular geometric characteristics and anisotropic mechanical demands of patient bone defect sites. Future design paradigms are poised to shift towards data-driven approaches. On one hand, future research can leverage deep learning algorithms to construct property–structure inverse design models. These models can automatically invert and generate non-linear, hierarchical gradient topological structures tailored to the specific stress distribution and mass transport requirements of the defect site. On the other hand, research can combine high-throughput experimental data to predict mechanical properties and cellular biological behaviors under different gradient features. This would drastically reduce trial-and-error costs prior to manufacturing, thereby enhancing the scientific rigor and success rate of the design.
(2) Four-Dimensional Printing-Driven Dynamic Adaptation and Spatiotemporal Programming: The regeneration of natural bone tissue is a dynamic evolutionary process accompanied by vascular ingrowth, matrix mineralization, and mechanical reinforcement. Future gradient scaffolds can introduce the time dimension by utilizing 4D printing technology to endow scaffolds with full-cycle dynamic adaptability. First, addressing the challenge of interface integration, future research can develop shape memory polymers (SMPs) sensitive to body temperature or physiological pH. This would enable scaffolds to undergo in situ deformation post-implantation, conformally filling irregular defect interfaces and creating mechanical interlocking, thus solving the clinical problem of poor bonding between rigid scaffolds and host bone. Second, to match the physiological rhythm of bone regeneration, future research can construct on-demand responsive gradient drug delivery systems. By designing the degradation characteristics of the material, the scaffold’s mechanical attenuation rate can be dynamically matched with the rate of new bone formation. Simultaneously, the stage-wise release of anti-inflammatory, angiogenic, and osteogenic factors can be achieved, realizing spatiotemporal gradient regulation synchronized with the physiological rhythm of bone regeneration.
(3) Systemic Regulation and Mechanism Elucidation of the Immuno-Osteogenic Coupling Microenvironment: Existing gradient designs mostly focus on directly inducing osteoblast differentiation, often overlooking the immune microenvironment during the initiating phase of bone regeneration. Future research must not only focus on constructing osteogenic gradients but also develop intelligent scaffolds with immunomodulatory gradients. By regulating micro/nano-topographical gradients or ion release gradients on the scaffold surface, an immune microenvironment favorable for vascularized bone regeneration can be established during the early healing stage. Furthermore, it is crucial to deeply elucidate how different gradient physical signals (e.g., mechanical gradients, pore size gradients) influence the communication and coupling between immune cells and osteoprogenitor cells through mechanotransduction pathways. This will provide a solid theoretical basis for designing more efficient pro-osteogenic gradient scaffolds.

## Data Availability

No new data were created or analyzed in this study. Data sharing is not applicable to this article.

## Abbreviations

The following abbreviations are used in this manuscript:

AM	Additive Manufacturing
ECM	Extracellular Matrix
TIPS	Thermally Induced Phase Separation
CFD	Computational Fluid Dynamics
DLP	Digital Light Processing
CAD	Computer-Aided Design
SEM	Scanning Electron Microscope
GelMA	Gelatin Methacryloyl
PCL	Polycaprolactone
PLA	Polylactic Acid
PEGDA	Poly(ethylene glycol) diacrylate
DMD	Digital Micromirror Device
PWM	Pulse Width Modulation
UV	Ultraviolet
NFES	Near-field Electrospinning
AI	Artificial Intelligence
G-code	Geometric Code
rBMSCs	Rat Bone Marrow Mesenchymal Stem Cells
TPMS	Triply Periodic Minimal Surface
GBR	Guided Bone Regeneration
HA	Hydroxyapatite
CT	Computed Tomography
MRI	Magnetic Resonance Imaging
CS	Chondroitin Sulfate
BPNN	Backpropagation Neural Network
FEA	Finite Element Analysis
CNN	Convolutional Neural Network
RGA	Regenerative Genetic Algorithm
EHD	Electrohydrodynamic
